# A Multi-Resident Number Estimation Method for Smart Homes

**DOI:** 10.3390/s22134823

**Published:** 2022-06-25

**Authors:** Andrea Masciadri, Changhong Lin, Sara Comai, Fabio Salice

**Affiliations:** Dipartimento di Elettronica, Informazione e Bioingegneria, Politecnico di Milano, 20133 Milano, Italy; changhong.lin@mail.polimi.it (C.L.); sara.comai@polimi.it (S.C.); fabio.salice@polimi.it (F.S.)

**Keywords:** multi-person detection, sensor data, smart environment

## Abstract

Population aging requires innovative solutions to increase the quality of life and preserve autonomous and independent living at home. A need of particular significance is the identification of behavioral drifts. A relevant behavioral drift concerns sociality: older people tend to isolate themselves. There is therefore the need to find methodologies to identify if, when, and how long the person is in the company of other people (possibly, also considering the number). The challenge is to address this task in poorly sensorized apartments, with non-intrusive sensors that are typically wireless and can only provide local and simple information. The proposed method addresses technological issues, such as PIR (Passive InfraRed) blind times, topological issues, such as sensor interference due to the inability to separate detection areas, and algorithmic issues. The house is modeled as a graph to constrain transitions between adjacent rooms. Each room is associated with a set of values, for each identified person. These values decay over time and represent the probability that each person is still in the room. Because the used sensors cannot determine the number of people, the approach is based on a multi-branch inference that, over time, differentiates the movements in the apartment and estimates the number of people. The proposed algorithm has been validated with real data obtaining an accuracy of 86.8%.

## 1. Introduction

The rapid development of the ICT sector has enabled scenarios where an ever-deeper interconnection between the physical and digital worlds is proposed (Phyigital—2007 by Chris Weil). One scenario is the home, where the interconnection between technologies and people enables the implementation of a paradigm for autonomous living, guaranteeing mutual safety (people, family members, and caregivers are in contact with each other) and allowing the identification of behavioral drifts and their subsequent compensation (behavioral drift is selectively compensated with solutions for the identified problem by containing costs and promoting personal autonomy) [1].

In recent years, applications of domiciliary technology systems interacting with the person have addressed issues such as activity recognition (e.g., [2,3,4]), health monitoring [5], security [6], and the prediction of future events [7].

The need to understand what is happening inside the dwelling requires that the phenomena to be tracked are observable; this assumes that there are suitable sensing systems (sensors and transducers) and that these are distributed more or less densely in the home. Considering the environmental sensing, the various proposals combine data collected from different types of sensors, such as RFIDs (Radio-Frequency IDentification), PIRs (Passive InfraRed) [8], contact sensors, pressure-sensitive mats [9], tilt sensors [10], power meters [11], inertial sensors, infrared array sensors [12], etc. In general, the types of sensor can be vision-based (e.g., cameras), wearable (generally based on inertial sensors like accelerometers, gyroscopes, etc.) or environment detection (e.g., motion or door/window sensors, temperature/humidity sensors, etc.). It is worth noting that both current regulations and perceived privacy violations make it difficult to use vision-based systems (2D and 3D cameras) over other detection techniques.

Considering wearable vs. non-wearable devices, a non-intrusive monitoring system (that is, without wearable devices) can guarantee a better trade-off between privacy and reliability because the absence of wearable devices eliminates or reduces some important critical issues, such as routine maintenance (e.g., recharging batteries) or the misuse of the device (e.g., taking it off in certain situations or forgetting to wear it). However, non-intrusive monitoring systems can provide reliable and direct measurements for many activities in a specific environment, such as room occupancy in the house, but do not achieve high levels of accuracy for many other tasks, such as calculating the number of people in a room. It is worth noting that in some scenarios, like in the case of aging, the exact calculation of the number of people in a home support system is not particularly relevant. What is often of interest is to detect whether the person is alone at home (and to inform the caregiver so that they pay more attention to the person) or whether there is a behavioral drift present, which leads the elderly to isolate themselves and progressively reduce their degree of sociality.

In these contexts, it is necessary to identify methodologies for identifying whether, when, and for how long the person is in the company of other people (possibly also considering the number).

People-counting in smart environments with distributed sensor networks has been studied in the past, but using a large number of sensors (e.g., up to 60 sensors distributed in a single apartment). Such a large number of sensors represents an important entry barrier for many households. The challenge is to tackle this task in sparsely sensorized apartments, with sensors that can be wireless (nowadays, many apartments are not equipped to host wired solutions) and can provide only local and simple information.

In this article, we propose a method that addresses technological problems (such as the blind times of the PIRs, their insensitivity in the absence of motion, and their different sensitivity depending on the distance and temperature of bodies), topological problems (such as possible sensor interference due to the inability to separate detection areas), and algorithmic problems. In the instrumented apartment, there is only one PIR per room and one on/off sensor on the front door. This is the minimum monitoring configuration: below this, one or more rooms are not ‘observable’. The house is modeled as a DAG (Directed Acyclic Graph). The model is used to deal with the fragmentation of the data stream that the various sensors can generate. In particular, we need to manage unwanted transitions between rooms when there are multiple people in the house. The DAG model can constrain the transition between adjacent rooms and avoid crossing walls. However, other issues for correct detection remain. The state of each room is represented by a set of values, one for each identified person. Each value decays over time and represents the probability that the person, while not specifying who they are, is still in the room. Because the sensors used in our setting cannot identify the number of people, the approach is based on multi-branch inference that, over time, differentiates the movements in the apartment and estimates the number of people; the limitation is that the number of people must be less than the number of rooms in the dwelling [13].

The main contributions of our work are:

- The method infers, step by step, the number of people in the house. It operates on non-ubiquity: if sensors are simultaneously active in rooms that cannot interfere, there are at least as many people as the number of active sensors. If the estimation is aimed at residents only, the method derives the exact number of people in the apartment. If the estimation is aimed at guests, the method gets whether the person is alone or whether there are more people.
- The method is based on a non-intrusive and minimum-cardinality sensors network with a single PIR sensor per room and a contact sensor on the front door. The scenario is typical of real-world situations.
- The method requires little scenario information (house map, sensor position, and the observability of each PIR) to determine both adjacency between rooms and interference situations between sensors.
- Finally, the method is unsupervised (can run in different scenarios without model training). This requirement is essential in order to make it possible to monitor a wide set of apartments.

The rest of the document is organized as follows. In Section 2, previous and related work is introduced. Next, Section 3 presents the proposed approach. In Section 4, the performance is evaluated. Finally, Section 5 discusses and concludes our work.

## 2. Related Works

During the years, various smart environment systems have been proposed. They have been used in many scenarios, such as family houses [14], offices [15], shopping malls [16], and museums [17], and have been applied for a variety of purposes, including tracking people in buildings [18], counting people numbers [19], recognizing human behavior [20], etc. In addition, energy consumption can be monitored, and the indoor environment can be controlled automatically by using appropriate sensors and controllers [21].

The types of detection means (e.g., through sensors or transducers) that can be used in smart environments are diversified. They can be roughly divided into two main categories, wearable devices and non-wearable devices, where the latter is in turn divided into sensors–transducers and multimedia-based devices. In terms of wearable devices, Bluetooth—BLE ([22,23]), UWB, Zigbee, WiFi, and RFID technologies [24], or specialized sensors (e.g., magnetic field sensors [25]) are widely used in indoor positioning systems to track or to localize people [26]. Sensors–transducers include various types of detection systems positioned in a smart environment to detect the movement of humans [27]. A non-exhaustive list of these devices includes Passive Infrared sensors [28,29], Thermal Sensors [30], and Force-Sensing Resistors (e.g., smart floors [31]. Moreover, pressure polymer, electromechanical film (EMFi), piezoelectric sensors, load cells, or WiFi can be used [31]. Finally, multimedia-based approaches can obtain rich context from the environment with videos [32] and audio [33,34,35].

Various approaches for people-counting in non-intrusive monitoring environments have been proposed without multimedia-based devices. Petersen et al. [36] propose an SVM-based method (Support Vector Machine) to detect the presence of visitors in the smart homes of solitary elderly because social activity is an important factor for assessing the health status of the elderly (social, psychological, and physical are, typically, the three dimensions of the health-related quality of life). Wireless motion sensors are installed in every room, and several key features are extracted and input to a SVM classifier, which is trained to detect multi-person events. The model has been validated with a two-subjects dataset and the results demonstrate the feasibility of visitor detection. The adopted method suffers from some criticalities: time is divided into fixed slots of time, with  epochs of 15 min; all possible room combinations are taken into account (i.e., n × (n − 1)/2 ) without considering that adjacent rooms could produce sensors interference; PIR blocking-time is not mentioned (blocking time could interfere with the activation order of PIRs); the time of the day is flagged a priori to consider the ‘circadian rhythm’; and finally, the approach is supervised.

Müller et al. [37] implement two approaches for inferring the presence of multiple persons in a test lab equipped with 50 motion sensors (data from CASAS) and some contact sensors; only two persons are monitored in the laboratory. One approach is a simple statistical method to derive the number of people based on the raw sensor data, while the second one uses multiple hypothesis tracking (MHT) and Bayesian filtering to track the people. The first method reaches an accuracy of 90.75%; the second one reaches 83.35%. The limit of this research is that the proposed method can only distinguish whether the house has a single person or multiple persons, but does not estimate the number of people. In addition, there are dense sensors in the test smart home, and the complex installation of sensors may limit the widespread use of such a system.

The authors of [38] estimate people numbers that satisfy the house topology and sensor activation constraints; then, a Hidden Markov Model is used to refine the result. The algorithm is validated in two smart homes and obtains high accuracy results when the smart home has 0 to 3 persons, but the accuracy decreases dramatically with 4 or more persons. In this work, both simulated and real data from different scenarios were used: ARAS (limitation: small rooms, few rooms, and only partially covered), ARAS-FC (limitation: few rooms without a dataset; the authors produced some data via simulation), and House 2 (limitation: the authors produced a simulated dataset). Unfortunately, it is not clear if they took into account the limits of the PIR sensors (for example, sensitivity and blocking time) and the criticality of the map; for instance, this could create interference between the different activations.

The work in [39] proposes an unsupervised multi-resident tracking algorithm that can also provide a rough estimation of the number of active residents in the smart home. They consider two datasets. The first is the dataset TM004 from CASAS, consisting of 25 ambient sensors distributed among eight rooms and with two-bedroom apartments with two older adult residents; occasionally, their child will come and stay in their house for a couple of days. The second is named Kyoto and contains a denser grid of sensors (91 sensors installed in six rooms—hallways included) with two residents; occasionally, they received friends for a visit of a few days. The algorithm has good performance, but this decreases as the number of residents increases; moreover, the algorithm tends to generate more resident identifiers when the same resident triggers the same sensor events, and it has a higher possibility of segmentation errors when tracking residents in a location where sensors are more densely deployed.

Other papers focus on the problem of the recognition of multi-resident activities in a smart-home infrastructure [40,41] (using the CASAS dataset with 60 sensors and 2 residents), [42] (using the ARAS dataset), and they can have as a consequence the possibility of counting the number of people. However, given their main goal, the number of sensors is typically very high, and the number of residents is limited to two persons.

A recent paper [43], focuses on the recognition of some daily activities in a multi-resident family home. The recognition of daily activities is a specialized task that requires to precisely identify the type of resident. For this purpose, authors used numerous and specialized types of actuators (e.g., a sensor module for a cup and a sensor box of the fridge) to distinguish the different activities performed by the individuals and using a data-driven and knowledge-driven combination method to recognize users. The article is very interesting, but, for the obvious reasons of observability of the phenomena, it requires a conspicuous number of transducers in addition to those normally used for home monitoring.

The survey in [27] focuses on the techniques for localizing and tracking people in multi-resident environments. For the counting problem, they identify three classes of approaches: (a) binary-based techniques based on binary sensors like PIRs—they typically exploit snapshots or, possibly, the history of snapshots with spatial and temporal dependencies to understand the number of people [44]; (b) clustering-based techniques that identify multiple non-overlapping clusters containing one or more targets; and (c) statistical-based techniques based on statistical models to estimate the number of persons.

The work in [45] aims at identifying visitors by using different measures of entropy for the cases with/without visitors in a smart home equipped only with PIR sensors and a door contact sensor that is used to confirm the visits and their duration. An accuracy of 98–99% is obtained in a setting where a single occupant typically resides in the home and a visitor arrives.

Alternative approaches to estimate the number of people have been proposed. For example [31] use WiFi: the movement can be detected through the analysis of the propagation effects of radio-frequency signals. Wang et al. [46] propose a method to count people by utilizing breathing traces, reaching 86% accuracy for four people. Similarly, in [47], Fiber Bragg Grating sensors are used for the detection and number of occupants, experimented with three people. Recent techniques also include voice recording to recognize up to three persons [48].

In the literature, vision-based methods have been always considered a reliable approach to estimate the number of people because the camera can obtain rich information. Vera et al. [49] proposed a system to count people using depth cameras mounted in the zenithal position: people are detected in each camera and the tracklets that belong to the same person are determined. Even though vision-based methods are efficient and reliable [14,50,51], they are unsuitable for smart homes due to privacy reasons. Algorithms based on wearable devices are infeasible for detecting visitors who do not wear such devices. Additionally, this intrusive method may not be acceptable for those people with low compliance.

Our algorithm avoids the usage of wearable devices and cameras: it adopts a system equipped with a very low number of presence detectors to realize non-intrusive monitoring, based on architectural modules of the BRIDGe project (Behavioral dRift compensation for autonomous and InDependent livinG) [52,53].

The proposed algorithm is based on minimal data about the house structure (plans of the flat) and sensor position, such as room adjacency and possible overlapping monitored areas (sensor interference), and can update the estimated number of people dynamically. The case study is with four inhabitants (a family with two adult children) in an apartment with a living room, a kitchen (open view), three bedrooms (a double room and two single rooms), two bathrooms, and a corridor. In the apartment, there are frequent guests, especially from Friday to Sunday (typically in the evening). The maximum number of people has reached six people. The apartment is instrumented with one PIR per room (eight PIRs) and a contact sensor on the main door. The PIR has a 2 s blocking time, 2 moves sensibility (the number of moves required for the PIR sensor to report motion), and a 12 s window time (the period of time during which the number of moves must be detected for the PIR sensor to report motion).

## 3. Proposed Method and Algorithm

### 3.1. System Architecture

The use-case scenario is a classical house with typical rooms: a kitchen, a living room, and one or more bedrooms and bathrooms. In each room, a PIR sensor is installed. PIR sensors are cheap and small, but they have some shortcomings: (a) the detection area of the PIR sensor is difficult to control, so that PIR sensors in different rooms may have overlapping areas of sensing range; (b) PIR sensors can only provide a binary response to the presence or not of people regardless of the number of people; (c) the sensitivity of the PIR is not uniform (it depends on the distance, the width of the visibility area—for example, edge zone or sectors of areas—and on the speed of the subject, on the characteristics of the subject [54]); and (d) the functioning of a PIR depends on a set of motion detection parameters (e.g., the blocking time).

Moreover, a contact sensor is installed at the entrance of the smart home. The sensor sends an activation signal when the door is opened. It is worth noting that a contact sensor (e.g., door and windows perimeter monitoring) is less critical, in terms of functioning and parameters, with respect to PIRs.

**Figure 1:**
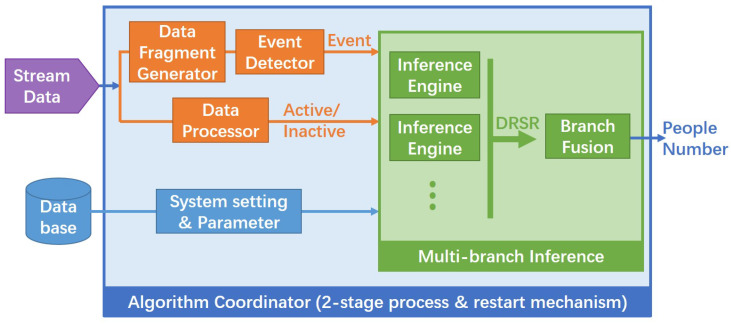
Architecture of the proposed algorithm.

Figure 1 shows an overview of the architecture and data flow of the proposed algorithm. The inputs of our algorithm are: the stream data from the PIR and contact sensors and some information stored in the database, including the house structure and the sensors settings. The stream data are processed by the Data Processor to detect the status of the sensors, which can be active or inactive. The Data Fragment Generator reads the sensor data and groups them into fragments concerning a continuous period that may represent interesting changes in the house. Then, the Event Detector detects events in the received data fragment, which may also include events coming from the door contact sensor. Based on the detected events, the status of the sensors, and the system setting, a multi-branch inference machine infers the number of people by fusing several independent inference engines that represent different possible scenarios compatible with the sequence of events. An algorithm coordinator controls all these modules and adds some functionalities that allow (a) to start the algorithm in any initial situation without any information about the number of residents and (b) to avoid accumulated errors that may occur in long-time runnings. All the modules are detailed in the next subsections.

### 3.2. Fragment Generation and Event Detection

#### 3.2.1. Data Fragment Generation

**Figure 2:**
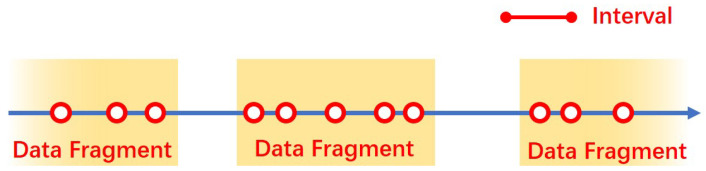
The generation of a data fragment: circles represent events that produce new data; if the time difference between two events is greater than interval, the new data are regarded as the beginning of a new data fragment.

Sensors produce and send data irregularly, depending on the activities that occur in the house. Typically, a series of signals are activated by the movement of a person within a small time interval. To recognize events that happen in the house, we divide stream data into semantic fragments composed of sequences of signals that occur in a certain interval as shown in Figure 2. Fragments are separated by periods that do not detect events for a given time interval.

In our Data Fragment Generator, only the active signal of PIR sensors and contact sensors installed at the entrance door are taken into consideration. Thus, data fragments represent events such as ‘somebody moves from the bedroom and goes out passing through the living room’.

#### 3.2.2. House Event Detection

The layout of the rooms in the house is modeled as a Directed Acyclic Graph (DAG) representing their adjacency. The algorithm works also in multi-floor buildings. The detection and inference of possible events is realized by finding the transition of active signals from generated data fragments.

**Figure 3:**
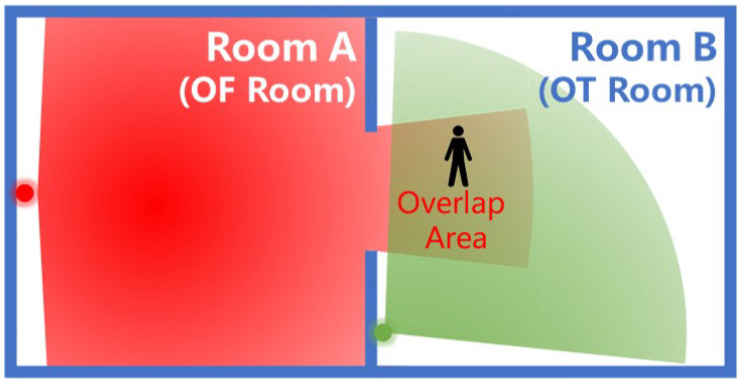
Overlap Example: Both sensors in Room A and B are active, but the overlap is in Room B, so this is defined as the *Overlapped True (OT)* room; instead, Room A is defined as the *Overlapped False (OF)* room.

Besides the movement of people between adjacent rooms, there are some special events:

- **Go in:** When someone enters the house, a ‘go in’ event happens. The total number of people in the house increases.
- **Go out:** Similarly, a ‘go out’ event happens when someone goes out of the house. In this case, the total number of people decreases. Notice that the exact number of people entering/exiting the house cannot be determined, so the algorithm must take into account this aspect.
- **Overlap:** The detection area of PIR sensors in different rooms may have overlapping detection areas. An example is shown in Figure 3. Such kinds of events need to be identified to have a better inference.

The overlap case depends on the direction and the installation place of sensors; therefore, the possible overlap areas can be identified in advance.

**Figure 4:**
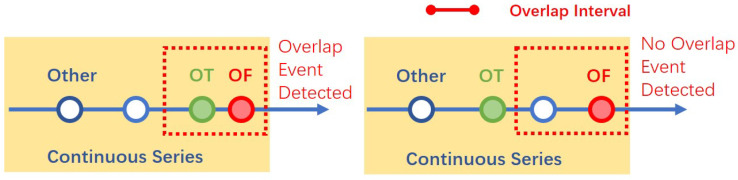
Overlap Event Detector: The last two signals highlighted in each data fragment are compared with the list of known overlap cases.

If an overlapping case occurs, both sensors are active: if the difference between their timestamps is less than a predefined *overlap interval*, the detector will detect an overlap event. Figure 4 shows the typical behavior.

### 3.3. House Status Estimation

#### Decayed Room Status Representation

To represent the house status, our method considers the following facts:

- PIRs transmit a state change when they detect a change in the infrared signals they receive. After an activation, PIR sensors remain inactive for a while and do not capture other events (blocking time). It is worth noting that PIR sensors do not change state when people are motionless.
- The latest data can be considered more reliable for the representation of the current status of the house compared to previous data.

To estimate the number of occupants accurately, besides the latest data, also the previous data need to be taken into account. In each room, more than one person may be present: the status of a room is represented by a set of values, one for each estimated person, that decay over time and that represent the probability that the persons are still in the room. We call this kind of representation *Decayed Room Status Representation ($DRSR_{t}$)* and the status of each person *j* in each room *i* at the time instant *t* *Room Status Signals ($RSS_{i,t}^{j}$)*. The value of each $RSS_{i,t}^{j}$ varies from 0 to 1: 1 means that the person has been detected, 0 that the person has left the room, an intermediate value that the person may be in the room.

**Figure 5:**
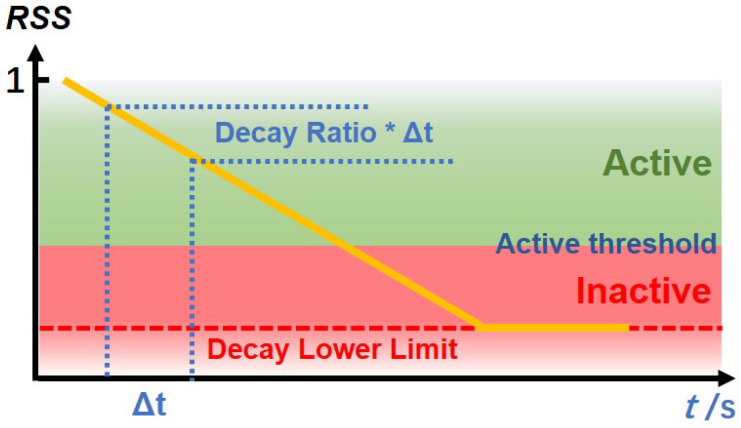
The graphical representation of $RSS_{i,t}^{j}$ with the active and inactive areas, the decay lower limit and the decay ratio.

Each $RSS_{i,t}^{j}$ decays over time with a given decay ratio until it reaches a lower limit, according to Equation (1), where $\Delta t_{i}$ is the time difference from the last update of the *i*-th room and $n_{i}$ is the number of persons estimated in the room.

(1) $$RSS_{i,t+\Delta t_{i}}^{j}=max\left\{RSS_{i,t}^{j}-decay\_ratio\times \Delta t_{i},decay\_lower\_limit\right\},j=1,2,...,n_{i}$$

where *decay_ratio* defines the decay speed of $RSS_{i,.}^{j}$ and *decay_lower_limit* is the limit that $RSS_{i,.}^{j}$ can reach. Such a decay mechanism can make up for the shortcomings of the PIR sensors that are insensitive to motionless people. When a person is detected in a room, if the adjacent room has not revealed an activity, we can assume that the person is still there. Therefore, the activation status can last for a certain period, until the $RSS_{i,t}^{j}$ value decays to the lower limit (*decay_lower_limit)*. Thus, from the status $DRSR_{t}$ of the house, we can determine whether a room is occupied or not by comparing their $RSS_{i,t}^{j}$ values with a given threshold. The description of $RSS_{i,t}^{j}$ is shown in Figure 5.

The $RSS_{i,t}^{j}$ also has the following tunable parameters:

- **Additional decay**: To balance the uncertainty between the case of motionless people (but still in the room) and people that have moved to other rooms, an additional decay value is defined to be added in case of inactivity signals.
- **Active threshold**: determines the status of a room. If an $RSS_{i,t}^{j}$ is higher than the active threshold, the room status is set as occupied; when it is lower than the active threshold, the person is removed from the counting for that room.

Because the transfer of people from one room to another can be detected from a data fragment as described in Section 3.2.2, if the algorithm detects a transfer from room A to room B, while room B already has one person in it, then the number of people in the status of that room will be set to 2. For example, if the active threshold is 0.2 and the RSSs in the house are those shown in Table 1, then the total number of people in the house is estimated to be equal to 3 because three $RSS_{i,t}^{j}$ values are greater than 0.2. Notice that for Room 2, two different $RSS_{2,t}^{j}$ values are available, one for each person.

### 3.4. Inference Engine

An inference engine is an entity that infers the status of the house. It has two attributes: the status of the rooms $RSS_{i,t}$ of the house described above and a confidence score which changes with the inferring process. The confidence score represents the consistency of the state of the house. Whenever an ambiguity condition occurs, the confidence score decreases to return to 1  when the ambiguity is resolved. For example, if the inference engine finds that a transfer from one room to another is concluded successfully, the inference process finishes, and the state of the house is updated. On the contrary, if some inconsistencies are found and the process needs to continue in order to solve these problems, the confidence score is decreased. The number of inference engines depends on the events in the house and the sensors data, as different branches for all the possible cases that could be inferred are generated.

When the algorithm starts, one inference engine is initialized with the confidence score set equal to 1. All rooms are regarded as empty, i.e., all $RSS_{i,t}$ are initially empty. When the system is running, the new sensors data and the events detected by the Data Fragment Generator are fed to the inference engine to update the status of the rooms. Then, the number of people is estimated based on the $RSS_{i,t}$ values.

The Inference Engine updates status of the rooms following the main rules below:

- ‘Go in’ event: For the room connected to the entrance door (for example, room *i* = 1), a new $RSS_{i,t}^{j}$ with value 1 is added and the status (number of people) is increased by 1. The event is identified through the analysis of the activation sequence between the input PIR and the ON/OFF sensor;
- ‘Go out’ event: For the room connected to the entrance door (for example, room *i* = 1), all $RSS_{i,t}$ values and the status are set to 0; if the room is currently estimated as empty (inconsistency state), the confidence is reduced.
- ‘Overlap’ event: the false activation signal is ignored;
- When the system receives an active signal, the algorithm determines if a room transfer has occurred according to the topology of the house. If a transfer happens, a new $RSS_{i,t}^{j}$ of the target room is set to 1. At the same time, the $RSS_{i,t}^{j}$ with the minimum value of the room where the person comes from is deleted. In case the active signal just intercepts an activity inside the room (person movement), all $RSS_{i,t}^{j}$ for that room are incremented through the following formula (in the current settings, *arise_ratio* is equal to the *decay_ratio*)
  (2) $$RSS_{i,t+\Delta t_{i}}^{j}=max\left\{RSS_{i,t}^{j}+arise\_ratio\times \Delta t_{i},1\right\},j=1,2,...,n_{i}$$
- If the PIR sensor does not activate for a certain time, but it may be possible that there are still persons in the room, the status of the room becomes inactive, and its $RSS_{i,t}^{j}$ values are decreased by the additional decay value.

#### Multi-Branch Inference

**Figure 6:**
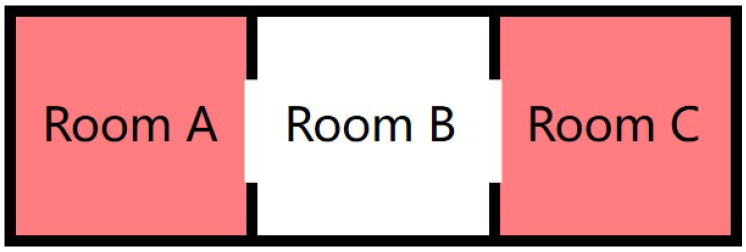
Example of transfer dilemma: One person is in Room A and another one is in Room C. If the PIR sensor in Room B is activated, it is hard to determine whether the person who activated it comes from Room A or Room C, until other events occur.

Because PIR and contact sensors cannot distinguish the number of people, we deal with this situation with a multi-branch inference approach to consider the context of sensors data. In some cases, the system may not be able to estimate the number of people accurately, like in the case where there are more than two candidate rooms that satisfy the room transfer condition, but after some inferences, the estimated result can finally converge to the ground-truth number. Figure 6 shows a simple example.

The maintenance of the proposed multi-branch inferring method is as follows:

- **Create Branch:** When a dilemma case occurs, several new inference branches are created for every possible movement case. Every branch has a confidence attribute representing its reliability. For example, for the transfer dilemma case in Figure 6, two inference engines are created—one for a possible transfer from Room A to Room B; another for the transfer from Room C to Room B, with independent room status values. Both continue to infer the house status simultaneously.
- **Merge Branch:** Branches that have the same status are merged. If two inference engines have the same status of the house for all the rooms, we regard them as the same inference engine, delete one of them, and sum their confidence scores.
- **Resize Confidence:** Confidence scores are scaled-up periodically according to Equation (3), where *confidence*${}_{i,t+\Delta }$ is the new confidence for engine *i*, ***confidence*** is the vector of all confidence values of the engines.
  (3) $$confidence_{i,t+\Delta }=\frac{1}{max(confidence)}\times confidence_{i+t}$$
- **Delete Branch:** To reduce the number of inference engines, all the confidences are sorted, and the inference engines with the lowest confidence values are deleted.
- **Branch Fusion:** All inference branches have different DRSR statuses and estimated people numbers. Specific methods are used to fuse the results of all the inference branches, such as voting, averaging, or weighted averaging based on branch confidence.

### 3.5. Algorithm

The general algorithm as well as all the steps described in the previous subsections are described in Algorithms 1–5.
**Algorithm 1** Main algorithm1:sensorId,sensorState,sensorTimestamp←getSensorEvent()2:**if**sensorState==On**then**3:    sensorsList←updateSensorsList(sensorID,sensorTimestamp,On)4:    **if** goIn(sensorsList,sensorID)==True **then**5:        **for each** DRSRinDRSRList **do**6:           $room_{entrance}\leftarrow addNewPerson\left(\right)$7:        **end for**8:    **else**9:        **if** goOut(sensorsList,sensorId)==True **then**10:           **for each** DRSRinDRSRList **do**11:               $room_{entrance}\leftarrow zeroPerson\left(\right)$12:           **end for**13:        **else**14:           **if** overlap(sensorsList,sensorId)==True **then**15:               DRSR←refresh(sensorsList,sensorId,time())16:           **else**17:               DRSR←update(sensorsList,sensorId,sensorTimestamp)18:           **end if**19:        **end if**20:    **end if**21:**else**22:    **if** sensorState==Off **then**23:        sensorsList←updateSensorsList(sensorID,sensorTimestamp,Off)24:        DRSR←update(sensorsList,sensorId,sensorTimestamp)25:        DRSR←rssDecay(sensorTimestamp)26:    **else**27:        DRSR←refresh(sensorsList,All,time())28:    **end if**29:**end if**

**Algorithm 2** Function goIn
1:

defgoIn(sensorsList,sensorId):boolean

2:
**if** (sensorId)==DOOR **then**
3:    **if** time(sensorsList.entrance)−time(sensorsList.door)≤DELTA **then**4:        returnTRUE5:    **else**6:        returnFALSE7:    **end if**8:
**end if**



**Algorithm 3** Function goOut
1:

defgoOut(sensorsList,sensorId):boolean

2:
**if** (sensorId)==DOOR **then**
3:    **if** time(sensorsList.door)−time(sensorsList.entrance)≤DELTA **then**4:        returnTRUE5:    **else**6:        returnFALSE7:    **end if**8:
**end if**



**Algorithm 4** Function update
1:

defupdate(sensorsList,sensorId,time):DRSR

2:
**if** dilemma(sensorsList,time,DAG)==True **then**
3:    DRSR←addNewDRSR(sensorsList,time)4:
**else**
5:    **if** roomsTransfer(sensorsList,time,DAG)==True **then**6:        DRSR←moveDRSR(sensorsList,time)7:    **else**8:        DRSR←refresh(sensorsList,sensorId,time)9:    **end if**10:
**end if**



**Algorithm 5** Function refresh
1:

defrefresh(sensorsList,sensorId,time):DRSR

2:

DELTA=time−lastTime

3:
**if** sensorId!=ALL **then**
4:    DRSR←rssArise(sensorsList,sensorId,time)5:
**else**
6:    **if** DELTA≥INTERVAL **then**7:         lastTime=time8:         DRSR←rssDecay(DELTA)9:         DRSR←resize()10:       DRSR←merge()11:       DRSR←fusion()12:       DRSR←delete(numDRSR)13:   **end if**14:
**end if**



### 3.6. Algorithm Coordinator

Two further important algorithm steps are introduced: the two-stage process and the restart mechanism. The former is needed to balance the accuracy and stability of our algorithm; the latter is used to avoid an accumulated error for long-time running.

There are two challenges that our algorithm has to face: the first one is that the initial number of people and the initial status of the house are unknown because the smart home system may start at any time; the second challenge is that sensors in the smart home cannot distinguish multiple persons. For example, if two people are in the same room, they are regarded as one person, because sensors cannot see the difference with respect to the case of a single person. In this condition, the algorithm would estimate fewer people. To solve the two problems above, the algorithm works in two different stages: refresh stage and stable stage.

- **Refresh stage:** When the system starts (the initial people number is set to 0) or when the entrance door opens (some people may come in or go out), the estimated people number is uncertain. The number of people is refreshed over time, and the estimated number can converge to a correct result. This process is realized by changing the lower limit of the room status. When this is set to 0, the estimated number can increase and also decrease.
- **Stable stage:** When the estimated number remains unchanged for a specific period, the lower limit of the room status is raised to a value that is greater than the active threshold, i.e., the estimated number can only increase. In this stage, our algorithm can perform a more stable estimation because people will not disappear when the door does not open.However, the algorithm should allow the increase in the estimated number because, in the refresh stage, some motionless/sleep inhabitants may have been ignored and the corresponding room statuses are falsely decaying to 0.

**Figure 7:**
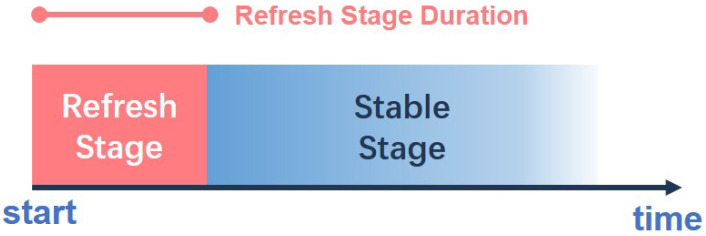
Diagram of the Two-stage Process: The algorithm starts, and after the Refresh Stage Duration, the algorithm switches to the stable stage.

A parameter called *Refresh Stage Duration* is set to switch the algorithm from the refresh stage to a stable stage. The diagram of our two-stage process is shown in Figure 7.

Because smart home systems need to run for months, our proposed algorithm also needs to run for a long time. To avoid accumulated errors for the house status and people number estimation, the algorithm needs to be restarted regularly. The length of the Refresh Stage Duration has been tuned on the field, and the results are shown in the experimental section.

## 4. Results

Our approach has been validated in a domestic environment equipped with smart sensors using the BRIDGe platform [52]. Data have been recorded for 14 days in a house with four people (a family with two adult children). In the apartment, there are frequent guests, especially from Friday to Sunday (typically in the evening). Six is the maximum number of people at a Saturday dinner (on the other days, the typical number is less or equal to four).

### 4.1. House Layout and Sensor Setting

**Figure 8:**
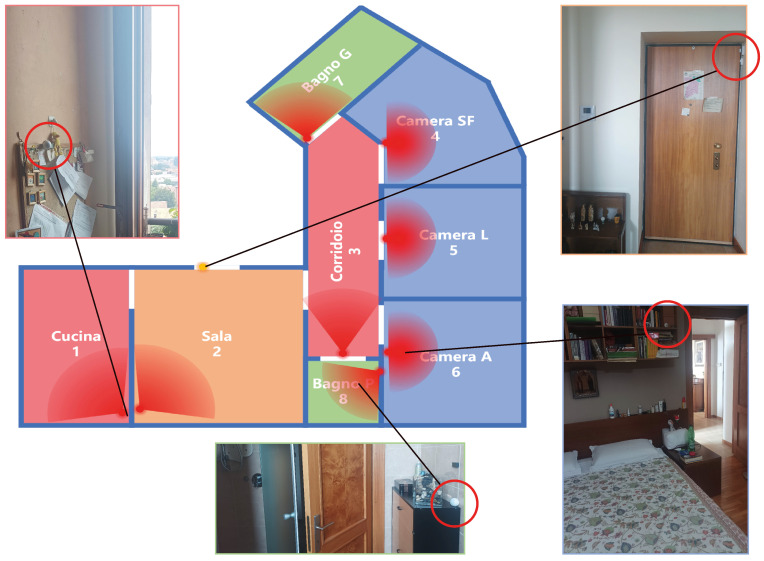
The Layout of Smart Environment with PIR sensors positions. The positions of the PIRs are highlighted with red circles in the pictures of the rooms. On the map represent, they are positioned in corresponding of the red circles, while the red sectors indicate the general direction of the sensors’ sensing areas, not the actual sensing range.

Figure 8 shows the layout of the house. It includes a kitchen (open view), a living room, two bathrooms, three bedrooms, and a corridor. The corridor connects bedrooms, bathrooms, and the living room. The entrance door of the house is in the living room.

The apartment is instrumented with one PIR per room (therefore eight PIRs) and a contact sensor on the main door. Data are collected by the FIBARO control unit (model HC2); all FIBARO sensors, Z-Wave protocol (868 MHz), are mesh networked to the FIBARO control panel. It is worth noting that the WiFi (there is a connection in the apartment) and Z-Wave connection do not interfere because they operate on different frequencies. Data transmission from the central unit to the cloud operates by events. Whenever a sensor changes state (the state—ON or OFF—and time in which the state change occurred), the record related to the sensor (SensorID, State, Time) is sent to the cloud. The PIRs are FIBARO Motion Sensors, type FGMS-001 (multi-sensor: PIR, vibration, temperature, and light), configured as follows: 2 s blocking time, 2 moves sensibility (number of moves required for the PIR sensor to report motion), and 12 s window time (period of time during which the number of moves must be detected for the PIR sensor to report motion). The PIRs are sensitive to direct sunlight. The PIRs were put in a condition not to be directly affected by the sun. There are no other particular and critical situations to take into account. The PIRS in the bathrooms are positioned far from water. By construction, they tolerate humidity; this characteristic is particularly important in Bathroom Small, where a shower is present and where it is possible to detect variations of 30% RH when taking showers in winter. Possible overlap cases exist in this smart environment as shown in Table 2. As described in Section 3.2.2, OT rooms are the correct rooms to be considered when the person is in the overlap area. For example, the first line indicates that when the person is in the corridor, there is an area where they may also be detected by the living room sensor.

There are four permanent residents, and they have private environments. In particular, there are two single rooms (Room person A and Room person L) and one master bedroom (Room persons F and S). Bathroom Big is used mainly by A, L, and F, while Bathroom Small is used mainly by S. In Bathroom Small, there is a shower used by all four family members. In the living room, there are two sofas and a television; there is no table. The table is only in the kitchen. For the ground truth, the arrival and leaving of people have been recorded manually, including in/out events, the change of people number, and the time when they were in or out. The time has been recorded manually and accurately (hours and minutes). The total people number changes when the door is opened; then, it may be distributed in different ways in the different rooms. Because the record of the contact sensor installed at the door is accurate to seconds, we used it to align the in/out time to reach second-level accuracy.

### 4.2. Indicators Design

To measure the performance of our approach, two kinds of indicators have been considered: the accuracy of the number of people and the stability of the number change.

#### 4.2.1. Accuracy Design

The accuracy represents the percentage of the time with a correct estimation with respect to ground truth. As shown in Equation (4), $T_{TotalTime}$ is the total time we measured, and the unit is in seconds.

(4) $$Accuracy=\frac{\sum_{i=0}^{n}TP_{i}}{T_{TotalTime}}$$

(5) $$T_{TotalTime}=t-t_{begin}$$

(6) $$T_{CorrectEstimate}+=\left[{\hat{n}}_{t}=n_{t}\right]\times \left(t-t_{prev}\right)$$

Accuracy is the overall accuracy of the validation dataset. $TP_{i}$ is the total time that the algorithm makes a correct estimation when the ground-truth number is *i*. $T_{TotalTime}$ stands for the total time from which the system begins to the current moment. *t* and $t_{begin}$ are the current timestamp and the moment when the system started, respectively. In the last equation, ${\hat{n}}_{t}$ is the estimated people number at the current moment, $n_{t}$ is the true people number, *t* is the current time, and $t_{prev}$ is the previous timestamp when the sensor data have been received.

#### 4.2.2. Stability Indicator Design

Stability can be represented by using the notion of information entropy. The information entropy is used to measure the uncertainty of inferred numbers. We take the last 10 min of the inference results to calculate the entropy because the earlier change of numbers may possibly be caused by people’s movements. The less the entropy, the better the system performance.

(7) $$Total\_Entropy=\frac{\sum Entropy(t)}{N}$$

(8) $$Entropy\left(t\right)=-\sum_{k=0}^{max({\hat{n}}_{t})}\left(p_{{\hat{n}}_{t}=k,t}ln\left(p_{{\hat{n}}_{t}=k,t}\right)\right)$$

(9) $$p_{{\hat{n}}_{t}=k,t}=\frac{\sum 1_{\hat{n}=k}}{T/F_{s}}$$

Total_Entropy is the average entropy of every moment. *N* is the total number of the received data from the validation dataset. Entropy(t) is the calculated entropy in the 10 min before time *t*. ${\hat{n}}_{t}$ is the estimated people number at the current moment, $p_{{\hat{n}}_{t}=k,t}$ is the probability that the algorithm estimates the people number is equal to k in the 10 min before time *t*, *T* is the sample period, and $F_{s}$ is the sample frequency; here, we set it to 1 Hz.

Notice that entropy alone is not enough to represent the stability. For example, if there are two people-counting results, such as (2,1,2,1,2,1) and (2,2,2,1,1,1), they have the same entropy value, but the former result is worse. Therefore, a measure of the frequency of changes in the estimated numbers is introduced, called Changecost(). The less the changes cost, the better the system performance.

(10) $$Total\_ChangeCost=\frac{\sum ChangeCost(t)}{N}$$

(11) $$ChangeCost\left(t\right)=\sum_{t=0}^{T}|{\hat{n}}_{t}-{\hat{n}}_{t-1}|$$

Total_ChangeCost is the average ChangeCost of the whole dataset. ChangeCost(t) is the calculated entropy in the 10 min before time *t*.

### 4.3. Experiment

The final selected parameters are shown in Table 3. The final accuracy result is 86.78% with about 36,000 sensors data from the eight PIRs and the contact sensors for 14 days.

Some examples of the parameter selections are reported next: the first example is the selection of the Refresh Stage Duration. We took values from 1 to 30 min and tested the data from the dataset and obtained the result in Table 4. It can be noticed that when the Refresh Stage Duration is 5 min, the algorithm can reach the highest accuracy of 91.78%, with acceptable values of the other indicators, such as Entropy and ChangeCost.

The next example concerns the selection of the Max Branch Number. Similarly, several values were tested to find out the best value. The results of the tests are shown in Table 5. From the table, we can see that when the Max Branch Number is set to 30, the algorithm obtains the best result.

Several ablation studies of the algorithm have been undertaken to compare the performance with different methods and settings.

First of all, the validation dataset has been tested without the multi-branch inference method, i.e., only one inference engine has been used to infer the status of the smart environment. As shown in Table 6, we can see that the multi-branch method has less Entropy and ChangeCost than the single-branch method, which means that the estimated result of the former method is more stable. Moreover, the Accuracy of the multi-branch method is higher than the single-branch method, by over 10%. Thus, the proposed multi-branch inference method plays an important role in our algorithm.

In the proposed algorithm, overlap events can be detected and this message can be used as information to help the inference engine infer the house status. By using this event detector, the shortcomings of PIR sensors can be remedied. To prove this, an ablation experiment has been conducted and the result is shown in Table 7. Additionally, the detection of door actions, including the ‘go in’ and ‘go out’ events, have also been taken into account. From Table 7, we can see that without detecting the ‘Overlap’ event, the algorithm obtained a worse result than the proposed method in all the indicators. The former method regards the false overlapping case of the PIR sensors as real activation signals, which leads to an incorrect inference of the house status. If the ‘Door Action’ event detector was forbidden, the algorithm failed to make a correct estimation because the door action is important for the house status inference.

### 4.4. Limitations of the Method

It is worth noting that to detect the right number of people in the apartment, the following conditions must hold:

1. The number of people in the apartment is lower than the number *n* of rooms with a PIR sensor (or to the number of PIR sensors that cover separate areas). If this is not the case, the algorithm will identify a number of people that is at most equal to *n*.
2. The blocking time of the PIR sensors reduces the accuracy of our algorithm, especially when residents move around quickly and/or frequently; the lower the value, the higher the accuracy of the proposed method. It is worth noting that the parameters of the sensors strongly depend on the technology (both for connectivity and detection), on the chipset, and on the available energy. The latter aspect is the predominant factor because liveness depends on energy consumption. For example, the Tellur WiFi motion sensor and Xiaomi Aqara Zigbee have a blocking time of 60 s, the FIBARO motion sensor Z-Wave—the type used in our case study—has a blocking time that varies from 2 to 8 s, while for wired sensors, the times are extremely lower, and also with mixed detection technology (e.g., the Risco BWare DT AM microwave in K band with PIR).
3. The dynamics of the in/out events from the apartment must be lower than the dynamics of the movements of the people in the house; if the stationary condition of the number of people in the rooms is long enough, the algorithm is more likely to identify the number of people in the apartment. In fact, as the time of the people staying in the apartment increases, the certainty of the results increases (if they move among different rooms).
4. People in the apartment do not always move in pairs. If the people move in groups, the algorithm will not distinguish them from the movement of a single person.

The proposed methodology is general and without any specific needs, excluding those reported above; the rooms are those of a typical apartment (kitchen, bedroom, bathroom, etc.), and the limitations are derived from the number of the rooms and their connections. A studio apartment, for example, is an environment that does not allow, in a non-intrusive way, to draw much information about the number of people (except for special ‘private’ events, such as the use of the bathroom). Although it is out of the scope of this article, in some real cases we have been faced with, by increasing the PIR densities in some specific rooms, they had a ‘complex’ characterization. For example, in an apartment with an open-space living area gathering, where there is a kitchen, dining table, living room, etc., the area has been virtually partitioned into sub-units in order to infer where people are moving and the type of activity they are doing. In these cases (with the limits reported above), it is also possible to estimate the number of people.

## 5. Conclusions

In this paper, we presented a people-number estimation algorithm based on non-intrusive, sparse-distributed sensors data from a multi-resident smart environment with work on the continuous flow of data generated by the sensors. Estimating the exact number of people in a family with more than two residents is a difficult task, especially in a sparse-distributed sensor network where each room has only one binary sensor to detect the presence of a human. However, the choice for such a setting, which is basic and minimal, is affordable in practice in many situations. Moreover, having a good, even if not precise, estimation of the number of people can be sufficient in many real scenarios of older people living alone at home.

Our algorithm has several advantages: it does not need to learn any data and therefore can be applied immediately, starting at any time, and only limited information about the house settings is needed. A good accuracy has been obtained thanks to the representation of the status of the rooms and the multi-branch inference based on the context.

As future work, we plan to also test other types of sensor data, such as bed/chair sensors, to evaluate the results in motionless situations [55] where no PIR sensors are activated.

## Figures and Tables

**Table 1:** Example of people occupation determination.

Rooms	Room 1	Room 2	Room 3	Room 4
RSS	[0.0]	[1.0, 0.3]	[0.15]	[0.73]
Status	Empty	2 people	Inactive	1 person

**Table 2:** Possible Overlap Cases in Validation Dataset.

Number of Case	OT Room of Case	OF Room of Case
1	Corridor	Living Room
2	Bedroom L	Living Room
3	Bathroom Big	Corridor

**Table 3:** Editable Parameters in the Algorithm.

Editable Parameters	Value	Editable Parameters	Value
Decay Ratio	0.003/s	Series Interval	40 s
Additional Decay	0.2/s	Door Action Interval	60 s
Active Threshold	0.1	Max Branch Number	50
Overlap Interval	10 s	Refresh Stage Duration	300 s

**Table 4:** Parameter Selection of Refresh Stage Duration.

Parameter Values	Accuracy	Entropy	ChangeCost
1 min	56.89%	0.155	0.820
3 min	88.63%	0.169	0.824
**5 min**	**91.78**%	**0.171**	**0.882**
10 min	89.12%	0.176	0.937
15 min	58.12%	0.213	1.375
20 min	12.17%	0.294	2.225
30 min	10.34%	0.325	2.894

**Table 5:** Parameter Selection of Max Branch Number.

Parameter Values	Accuracy	Entropy	ChangeCost
10	49.04%	0.196	1.093
20	73.50%	0.212	1.430
**30**	**84.21**%	**0.177**	**1.002**
40	81.14%	0.177	0.952
50	81.14%	0.177	0.952
60	83.78%	0.173	0.913
70	81.14%	0.174	0.884

**Table 6:** Comparison of multi-branch method and single-branch method.

Method	Accuracy	Entropy	ChangeCost
Multi-branch Inference	86.785%	0.152	0.757
Single-branch Inference	75.695%	0.160	0.785

**Table 7:** Ablation Study on Event Detection.

Method	Accuracy	Entropy	ChangeCost
Proposed Method	86.78%	0.152	0.757
Without ‘Overlap’ Detector	55.71%	0.172	0.904
Without ‘Door Action’ Detector	fail	fail	fail

## Data Availability

The data presented in this study are available on request from the corresponding author. The data are not publicly available due to privacy reasons.
